# Stability Studies of Amorphous Ibrutinib Prepared Using the Quench-Cooling Method and Its Dispersions with Soluplus^®^

**DOI:** 10.3390/polym16141961

**Published:** 2024-07-09

**Authors:** Igor Mucha, Bożena Karolewicz, Agata Górniak

**Affiliations:** 1 Department of Basic Chemical Sciences, Wroclaw Medical University, Borowska 211 A, 50-556 Wroclaw, Poland; igor.mucha@umw.edu.pl; 2 Department of Drug Form Technology, Wroclaw Medical University, Borowska 211 A, 50-556 Wroclaw, Poland; 3 Laboratory of Elemental Analysis and Structural Research, Wroclaw Medical University, Borowska 211 A, 50-556 Wroclaw, Poland; agata.gorniak@umw.edu.pl

**Keywords:** ibrutinib, amorphous form, quench-cooling method, amorphous solid dispersion, soluplus, stability, solid-state characterization

## Abstract

The successful development of an amorphous form of a drug demands the use of process conditions and materials that reduce their thermodynamic instability. For the first time, we have prepared amorphous ibrutinib using the quench-cooling method with very high process efficiency. In the presented study, different formulations of amorphous active pharmaceutical ingredient (API) with Soluplus (SOL) in various weight ratios 1:9, 3:7, and 1:1 were prepared. The obtained samples were stored under long-term (25 ± 2 °C/60%RH ± 5% RH, 12 months) and accelerated (40 ± 2 °C/75%RH ± 5% RH, 6 months) storage conditions. The physical stability of amorphous ibrutinib and ibrutinib–Soluplus formulations was analyzed using differential scanning calorimetry (DSC), thermogravimetric analysis (TGA), powder X-ray diffraction analysis (XRPD), Fourier transform infrared spectroscopy (FTIR), and scanning electron microscopy (SEM). The lack of significant interactions between the ingredients of the formulation was confirmed by FTIR analysis. An increase in moisture content with an increasing SOL weight ratio was observed under accelerated aging and long-term conditions. Additionally, a slight increase in the moisture content of the stored sample compared to that at the initial time was observed. The results revealed the physical strength of the polymeric systems in the presence of high humidity and temperature. The observed high thermal stability allows the use of various technological processes without the risk of thermal degradation.

## 1. Introduction

To date, there are a variety of techniques used for the preparation of amorphous solid active pharmaceutical ingredients (API), including the quench-cooling method, freeze-drying and spray-drying method, milling method, wet granulation method, and drying of solvated crystals method [1]. The mechanism for the transformation of the crystalline to its amorphous form involves direct conversion caused by mechanical activation in the milling process or intermediate transformation to a thermodynamically stable non-crystalline form and the thermodynamically unstable amorphous solid via the utilization of the quench-cooling method [2]. The differences in the thermal, structural, and physical properties of various amorphous forms of the same drug, also known as “pseudopolyamorphism”, confirm that molecular mobility is crucial for the recrystallization process but is also dependent on the manufacturing technique [3]. Reports have described the obtainment of different physicochemical properties and stable amorphous forms of simvastatin [4], indomethacin [2], griseofulvin [5], glibenclamide [6], dipyridamole [7], and carbamazepine [8]. Analysis of amorphous simvastatin produced by cryo-milling and melting and quench cooling showed that the cryo-milled samples had lower physical stability (20 h at 20 °C/35%RH) compared to samples prepared by quench cooling (1 month at 20 °C/35%RH) [4]. Additionally, the Raman spectra of the amorphous form of cryo-milled simvastatin and the quench-cooled form were different. The thermodynamic parameters showed that the cryo-milled form was more ordered than the quench-cooled form [4]. Chikhalia et al. showed that the ball milling process generated particles with an amorphous surface and the incomplete transformation of the inner core. These particles could contain more nucleation seeds for recrystallization than the melted and quench-cooled samples [9]. Another report showed that amorphous indomethacin could be obtained using six different methods, where the samples were ranked depending on their stability: quench-cooled samples > cryo-milled (α-form) > spray dried > ball milled (α-form) > ball milled (γ-form) = cryo-milled (γ-form) [2]. The onset of crystallization of amorphous indomethacin produced by melting and quench cooling was reported after approx. 25 days when stored at 25 °C. Furthermore, 50% of the amorphous indomethacin was converted to its crystalline counterpart within 8 days when stored at 20 °C under dry conditions [4]. Wojnarowska et al. described the use of quench-cooling of the melt and cryogenic grinding for the preparation of glassy glibenclamide. The obtained quenched glibenclamide sample was stable over a wide temperature range, while the cryo-milled glibenclamide crystallized easily at 373 K. During 210 days of storage of both samples under dry conditions at room temperature, crystallization was not observed [6].

The main problem with the amorphous state is its physical instability during aging in the form of phase separation and recrystallization, so improved strategies for the stabilization of amorphous compounds in pharmaceutical development are still needed [10,11,12]. The risk of recrystallization of an amorphous material occurs during the typical shelf life of a drug (approx. 2 years), limiting the benefits of amorphous formulation development [13]. Current research on the stabilization of amorphous solids focuses on the prevention of crystallization via selecting the appropriate storage conditions in which amorphous solids are stable and the addition of excipients [1,14]. The excipients for the amorphous dispersion must be carefully selected to optimize the solubility of the API in the polymer and reduce the risk of phase separation in the amorphous system [15]. Soluplus^®^, an amorphous polyvinyl caprolactam-polyvinylacetate-polyethylene glycol graft copolymer, is a bifunctional excipient, polymer carrier, and active solubilizer, which can be used as the carrier of the fourth generation of solid dispersions [16,17]. Reported stability tests (60% RH/25 °C) revealed that dispersions of sulfonamides prepared with Soluplus^®^ by the ball milling method remained dry and powdery under high humidity storage conditions. In turn, dispersions with PVP storage under the same conditions form a sticky paste in less than 2 weeks, which highlights the advantage of using Soluplus^®^ as a pharmaceutical excipient, increasing physical stability [15].

Ibrutinib is the first Burton tyrosine kinase inhibitor in its class, currently being applied to the treatment of B-cell cancers [18]. Commercial medical products with poor water-solubility from II BCS class ibrutinib are available in the form of capsules and tablets containing doses in the range of 70–560 mg. Considering the absolute bioavailability of ibrutinib is only approx. 3% after oral administration. Hence, the development of formulations containing amorphous forms of this API and its dispersions with Soluplus as a solubilizing excipient is required for new dosage forms in formulation design [19]. Simoes et al. prepared an amorphous form of ibrutinib but failed to demonstrate its physical stability, where it recrystallized after 1 month of stability at 40 °C/75% RH and after 6 months at 25 °C/60%RH. To stabilize the described amorphous form, a solid dispersion of IBR-Soluplus was prepared by hot-melt extrusion, maintaining drug stability for 6 months under long-term and accelerated conditions [10]. This indicated that the molecular mobility of the IBR compound in the prepared matrixes was slow enough to avoid crystallization, even when stored under accelerated conditions. Since the current literature demonstrates that the differences in the properties of amorphous materials depend on the preparation method [20,21], it is important to examine the effect of the quench-cooling preparation method on the physicochemical properties and physical stability of the amorphous form of ibrutinib and its solid dispersions with Soluplus^®^.

## 2. Materials and Methods

### 2.1. Materials

**Figure 1:**
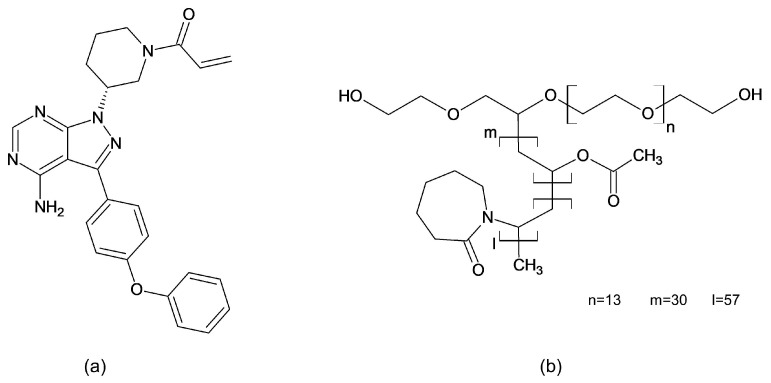
Chemical structure of ibrutinib (**a**) and Soluplus^®^ (**b**).

Crystalline ibrutinib form A (purity > 95%, CAS 936563-96-1) (Figure 1a) was supplied by abcr GmbH (Karlsruhe, Garmany). Soluplus^®^ (Figure 1b) was provided by BASF SE (Ludwigshafen, Germany).

### 2.2. Preparation of Amorphous Ibrutinib and Formulation with Soluplus

Amorphous ibrutinib was prepared by quench-cooling the crystalline form of ibrutinib. Briefly, 10 g of the commercial crystalline form of ibrutinib was melted and mixed in a quartz tub under a nitrogen atmosphere in an electric furnace at 180 °C for 30 min. It was then quench-cooled by placing the tub in a beaker containing liquid nitrogen for 15 min. Three different formulations of amorphous ibrutinib with Soluplus^®^ that varied in weight ratios 1:9, 3:7, and 1:1, respectively, were prepared. The accurately weighed samples of amorphous ibrutinib were mixed with the polymer using a mortar and pestle to form homogeneous mixtures and finally sieved using an 80 μm sieve.

### 2.3. Physical Stability Studies

The physical stability of amorphous ibrutinib and ibrutinib–Soluplus formulations was determined using differential scanning calorimetry (DSC), thermogravimetric analysis (TGA), powder X-ray diffraction analysis (XRPD), Fourier transform infrared spectroscopy (FTIR), and scanning electron microscopy (SEM) analysis. The prepared samples were stored in Eppendorf tubes under defined conditions in two BINDER environmental simulation chambers, 25 ± 2 °C/60%RH ± 5% RH (long-term conditions) and 40 ± 2 °C/75%RH ± 5% RH (accelerated conditions), respectively. The long-term stability studies were conducted periodically over 12 months (directly after preparation—day 0, 1 week, 2 weeks, 1 month, 2 months, 3 months, 6 months, and 12 months). Testing under the accelerated storage conditions was conducted periodically over 6 months (directly after preparation—day 0, 1 week, 2 weeks, 1 month, 2 months, 3 months, and 6 months). The samples were described as follows: crystalline ibrutinib form A (IBR raw), amorphous IBR at initial time (IBR_0), amorphous IBR stored for 6 months (IBR_acc), amorphous IBR stored for 12 months (IBR_long), IBR:SOL 1:1 at initial time (IBR:SOL 1:1_0), IBR:SOL 1:1 stored 6 months (IBR:SOL 1:1_acc), IBR:SOL 1:1 stored for 12 months (IBR:SOL 1:1_long), IBR:SOL 3:7 at initial time (IBR:SOL 3:7_0), IBR:SOL 3:7 stored for 6 months (IBR:SOL 3:7_acc), IBR:SOL 3:7 stored for 12 months (IBR:SOL 3:7_long), IBR:SOL 1:9 at initial time (IBR:SOL 1:9_0), IBR:SOL 1:9 stored for 6 months (IBR:SOL 1:9_acc), IBR:SOL 1:9 stored for 12 months (IBR:SOL 1:9_long), Soluplus^®^ at initial time (SOL_0), Soluplus^®^ stored for 6 months (SOL_acc), and Soluplus^®^ stored for 12 months (SOL_long).

### 2.4. Differential Scanning Calorimetry (DSC)

Thermal properties were analyzed using a DSC 214 Polyma instrument (Netzsch, Selb, Germany) equipped with an Intracooler IC70 (Netzsch, Selb, Germany). The accurately weighed samples (4.0 mg ± 0.1 mg) were studied using a heat-flow measurement, with nitrogen at a flow rate of 25 mL/min as the purge gas. The samples were placed in sealed standard aluminum pans (25 µL) with perforated lids. An empty pan of the same type was employed as a reference. The measurements were recorded at a heating rate of 10 °C/min in the temperature range of 0–250 °C. The DSC instrument was calibrated using 6 melting standard samples from a calibration set of 6.239.2–91.3 supplied by Netzsch. Netzsch Proteus Analysis software version 7.1.0 package was used for data analysis.

### 2.5. Thermogravimetric Analysis (TGA)

Thermal stability was investigated using a TG 209 F1 Libra (Netzsch, Selb, Germany). Samples (approx. 4.0 mg ± 0.1 mg) were weighted into alumina pans (150 μL) and heated from room temperature to 850 °C at a heating rate of 10 °C/min under a nitrogen atmosphere (flow rate of 25.0 mL/min). For each sample of ibrutinib, polymer, or ibrutinib–Soluplus formulation, a minimum of two measurements was performed to establish reproducibility. Netzsch Proteus Analysis software version 7.1.0 was used to determine the mass change and DTG curves.

### 2.6. Powder X-ray Diffraction Analysis (XRPD)

Diffractograms of all samples were recorded at ambient temperature on a Bruker D2 PHASER diffractometer (BRUKER AXS, Karlsruhe, Germany) with a LynxEYe detector using Cu Kα1,2 radiation (1.5418 Å). The diffraction data were collected in the Bragg-Brentano (*θ*/2*θ*) horizontal geometry between 5° and 35° (2*θ*) with a step size of 0.02° (2*θ*) and time 0.5 sec/step. The optics of the D2 PHASER diffractometer was a system of Soller slit modules with 2.5°, a divergence slit with 0.1 mm, 1.0 mm anti-scatter shields, and a Ni filter. The X-ray tube voltage and current were pre-set to 30 kV and 10 mA, respectively. Diffrac. EVA software (version 2.1., BrukerAXS, Karlsruhe, Germany) was used to evaluate X-ray diffraction data.

### 2.7. Fourier Transform Infrared Spectroscopy (FTIR)

FTIR spectra were recorded at room temperature using a Nicolet 380 FTIR spectrometer (Thermo Scientific, Waltham, MA, USA) equipped with an attenuated total reflectance (ATR) attachment. The spectra were collected over a frequency range of 400 cm^−1^ to 4000 cm^−1^ at a resolution of 4 cm^−1^. The commercially available software OMNIC version 5.0 (Thermo Scientific, Waltham, MA, USA) was used for the data acquisition and spectral analysis. The spectrum of each sample was an average of 128 respective scans, with three spectra taken per aliquot. Background readings were collected and subtracted from each spectrum before data output.

### 2.8. Scanning Electron Microscopy (SEM)

A scanning electron microscope, Zeiss EVO MA 25 (Carl Zeiss NTS GmbH, Oberkochen, Germany), equipped with a backscattered electron detector (BSD), was used to assess the particle morphology. The samples were sputtered with a thin layer of gold and palladium (60:40; sputter current 40 mA; sputter time 50 s) using a Quorum machine (Quorum International, Fort Worth, TX, USA). The accelerating voltage was set to 20 kV, and working distance was ~4–7 mm.

## 3. Results and Discussion

The quench-cooling method used in the presented research is a rarely reported technique for the development of amorphous drugs. However, in contrast to other techniques, volatile organic solvents and sophisticated equipment are not required, only careful temperature monitoring. The limitation of this method is that it is only for substances that melt without decomposing. Additionally, the quench-cooling method is characterized by very high process efficiency. In this work, 95% efficiency was obtained, which is practically unattainable with other advanced methods, such as milling, hot-melt extrusion, or spray drying.

In the quenching cooling method, the lattice structure of the crystalline drug is destroyed and transformed into a liquid state. Then, the drug is rapidly cooled using a supercooled liquid without crystallization, thus producing a drug in an amorphous state. According to the literature, the poor stability of amorphous forms obtained by milling and spray-drying methods compared to our proposed quenching cooling method may be due to highly defective crystals and nucleation residues in amorphous forms. The risk of recrystallization increases during storage because the nucleation residues cause further crystallization and growth of crystals. The spray-drying method, in turn, ensures relatively quick evaporation of the solvent, resulting in the formation of hollow and porous particles whose large surface promotes the amorphous drug to be more susceptible to recrystallization [22].

Owing to the issue of physical instability of drugs in an amorphous state during aging, leading to phase separation and its recrystallization, we examined the stabilization of amorphous ibrutinib–Soluplus polymer. Amphiphilic polymers, such as Soluplus, act simultaneously as matrix-forming polymers. Hence, they have advantages over traditional polymers for improving drug dissolution and absorption [23], displaying superior solubilizing effects for BCS class II drugs, but also forming micelles in aqueous solution [24]. Additionally, selecting a polymer from a biopharmaceutical perspective is attractive because of its advantageous properties, including minimum toxicity, low hygroscopicity, low glass transition temperature, and good thermal stability [25]. Moreover, it can greatly increase the wettability of a drug that is highly dispersed in amorphous solid dispersions (ASD) and inhibit the precipitation or crystallization during dissolution. Soluplus^®^ also prevents the aging of ASD during storage to a certain extent by forming hydrogen bonds with the drug [26].

In our study, amorphous ibrutinib (IBR) was obtained using the quench-cooling method, and different formulations of amorphous solid dispersions with Soluplus^®^ (SOL) in various weight ratios 1:9, 3:7, and 1:1, respectively, were prepared. Samples were stored under long-term (25 ± 2 °C/60%RH ± 5%) and accelerated (40 ± 2 °C/75%RH ± 5% RH) storage conditions. The physical stability of IBR and ibrutinib–Soluplus formulations was analyzed by DSC, TGA, XRPD, FTIR, and SEM.

### 3.1. Thermal Analysis

**Figure 2:**
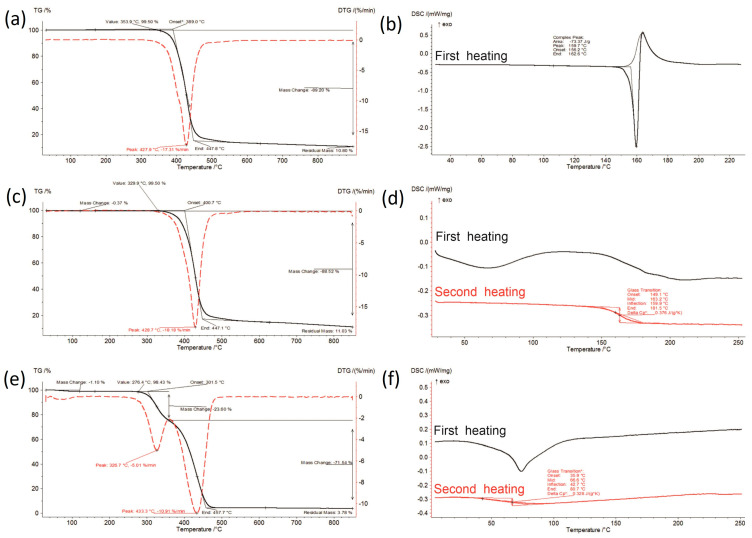
Thermal analysis of initial materials: (**a**) TG curve of IBR raw, (**b**) DSC curve of IBR raw, (**c**) TG curve of IBR_0, (**d**) DSC curve of IBR_0, (**e**) TG curve of SOL_0, and (**f**) DSC curve of SOL_0.

Thermal characteristics of the initial materials—raw (crystalline) IBR, amorphous IBR, and SOL—were determined using DSC, TG, and DTG (Figure 2). The thermal decomposition parameters were as follows: temperature of maximum weight loss rate (T_m_); extrapolated onset temperature of decomposition (T_onset_); rate of mass loss, which corresponds to T_m_; extrapolated temperature at which the degradation process ends (End*).

**Figure 3:**
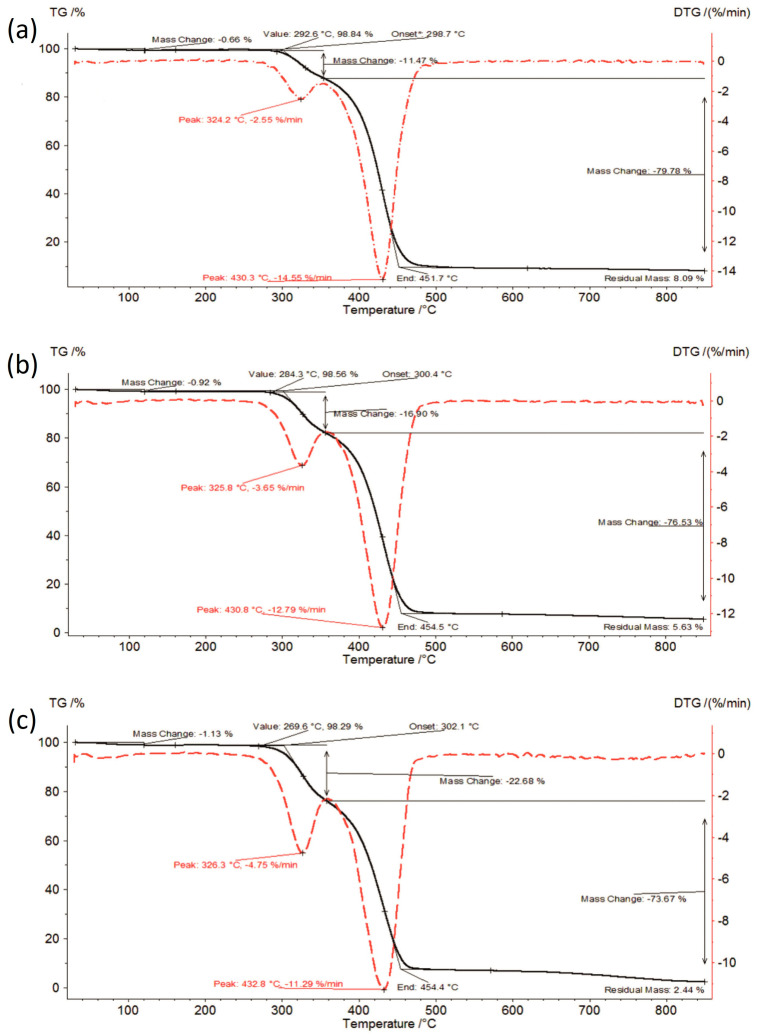
TG curves of the investigated formulations at initial time: (**a**) IBR:SOL 1:1_0, (**b**) IBR:SOL 3:7_0, and (**c**) IBR:SOL 1:9_0.

**Figure 4:**
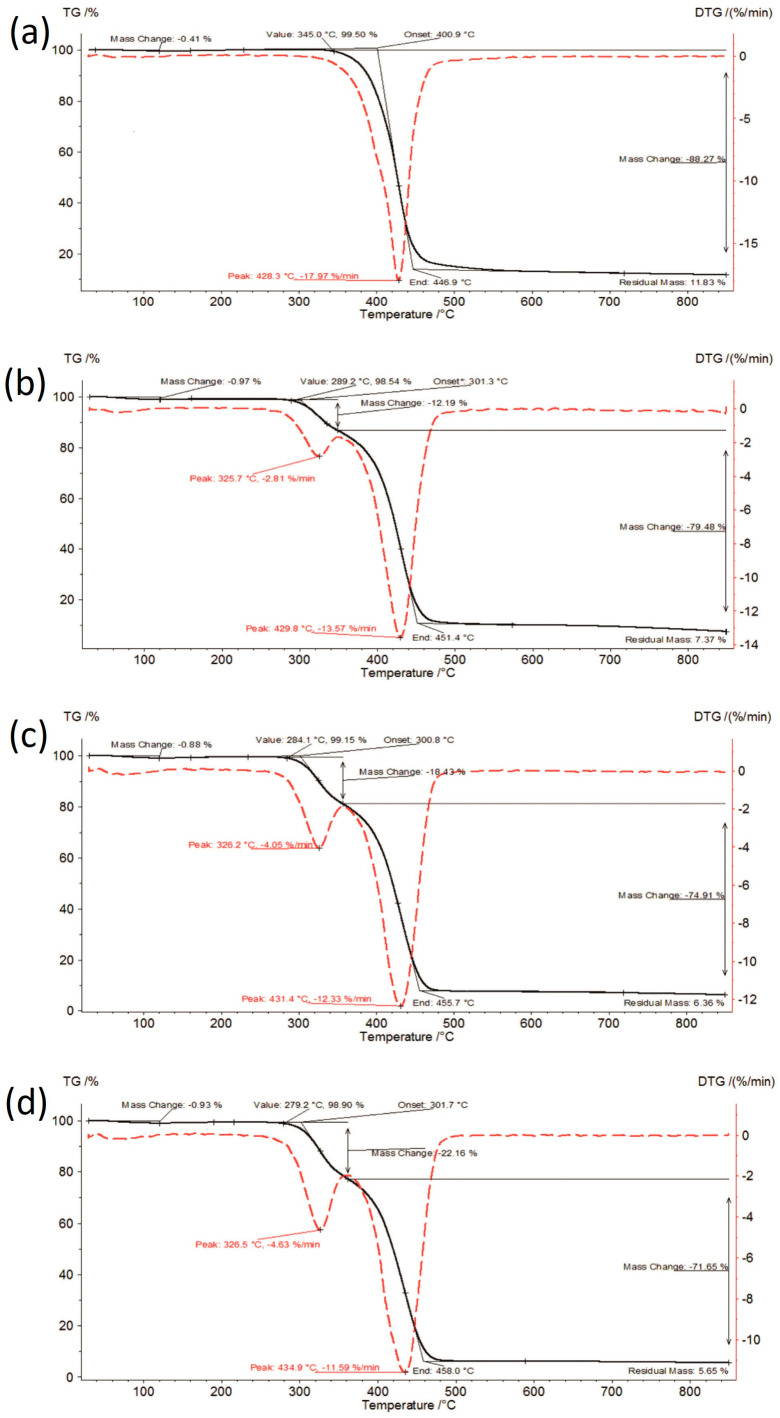
TG curves of samples stored for 6 months under accelerated conditions: (**a**) IBR_acc, (**b**) IBR:SOL 1:1_acc, (**c**) IBR:SOL 3:7_acc, and (**d**) IBR:SOL 1:9_acc.

**Figure 5:**
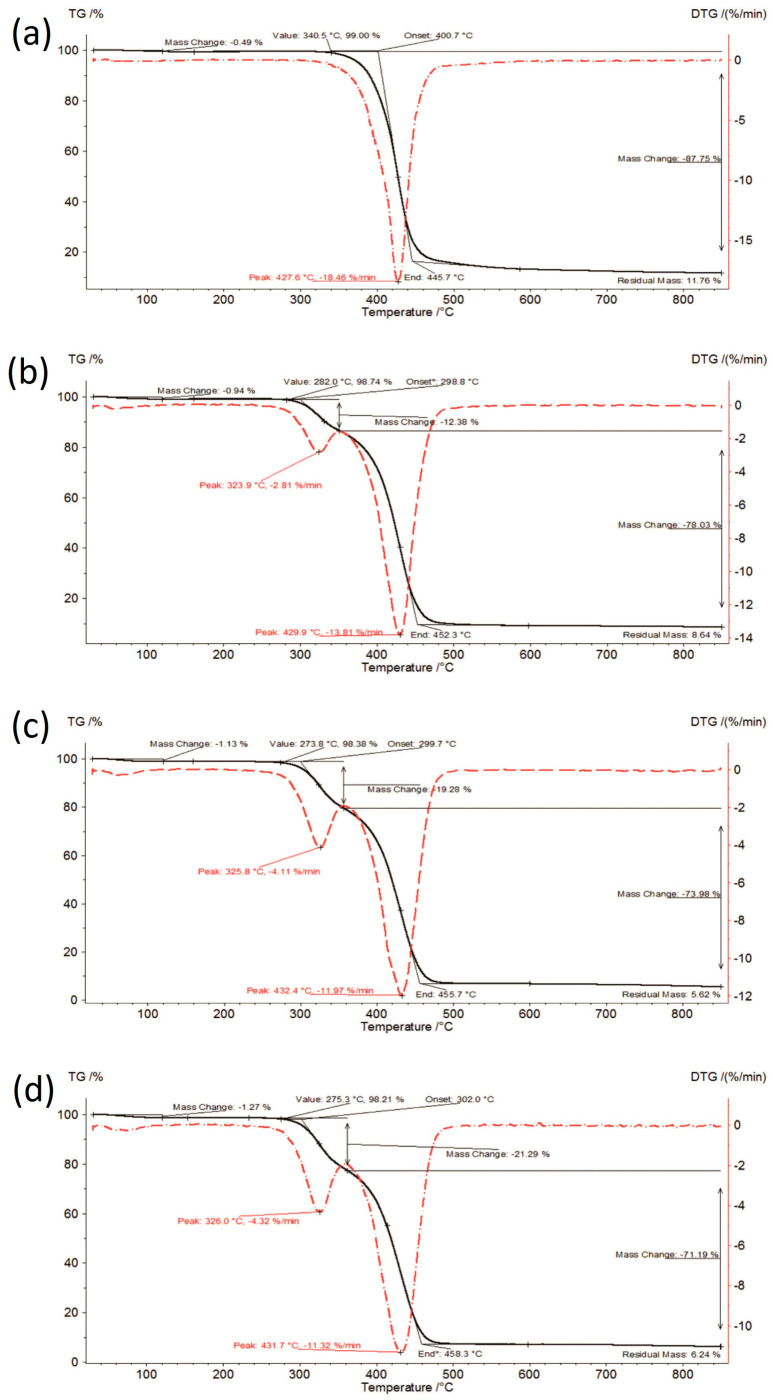
TG curves of samples stored for 12 months under long-term conditions: (**a**) IBR_long, (**b**) IBR:SOL 1:1_long, (**c**) IBR:SOL 3:7_long, and (**d**) IBR:SOL 1:9_long.

TG curves of the investigated formulations at initial time, stored for 6 months under accelerated conditions and stored for 12 months under long-term conditions are shown in Figure 3, Figure 4 and Figure 5.

**Figure 6:**
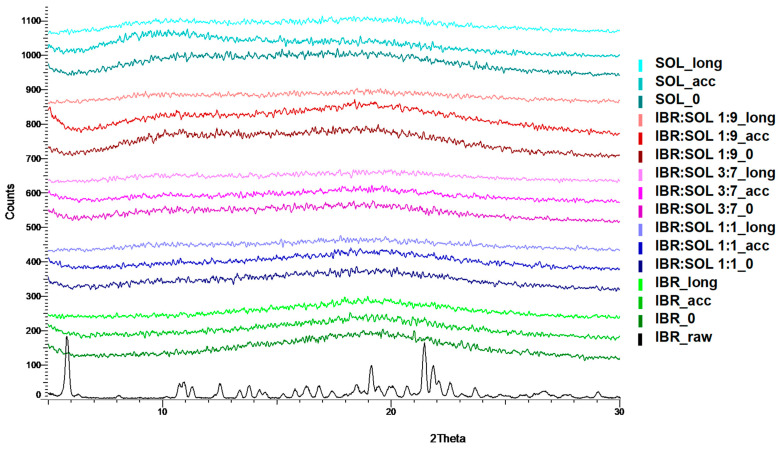
XRPD patterns of raw and amorphous IBR, Soluplus^®^, and IBR:SOL formulations at initial time and after stability tests.

The starting material was commercial ibrutinib in crystalline form, which was confirmed by DSC. The DSC curve showed only one sharp endothermic peak corresponding to the melting of the crystalline IBR at a temperature of 156.2 °C (T_onset_) and a value of the melting enthalpy of 73.37 J/g. The obtained results were consistent with the literature data [18,27] and indicated the presence of the most thermodynamically stable crystalline form A. The absence of thermal effects up to 100 °C and the lack of mass loss (Figure 2a) indicated that the anhydrous form of IBR was present. Crystalline IBR was thermally stable up to approx. 353.9 °C, above which the sample underwent one stage of decomposition (one mass loss was visible in the TGA curve and one signal on the DTG curve). The obtained diffractograms confirmed the presence of a crystalline form A (Figure 6), which was consistent with the literature [27].

Amorphous ibrutinib was prepared using the quench-cooling method with crystalline IBR. During cooling and reheating, a step change of heat flow in the DSC curve was observed, and the lack of a sharp endothermic peak characteristic of the crystalline form indicated the amorphization of the sample (Figure 2d). The glass transition temperature of 163.2 °C and ΔCp = 0.376 J/gK was determined. Additionally, the broad endothermic effect up to 100 °C during the first heating indicated the presence of 0.37% moisture (Figure 2c). The diffractogram exhibited a broad halo in the wide-angle region of the tested sample (Figure 6), which also confirmed its amorphous state. The SOL used to obtain the formulation was amorphous, which was confirmed by DSC (Figure 2f) and XRPD (Figure 6). This polymer was stable up to 276.4 °C, decomposed in a two-stage process, and contained approx. 1.1% moisture, which was visible in the TG curve (Figure 2e) and the DSC curve (Figure 2f). The glass transition temperature of the polymer was determined at 66.6 °C (Figure 2f).

The thermal analysis results are presented in Figure 2, Figure 3, Figure 4 and Figure 5 and summarized in Table 1. Amorphous IBR contained the least amount of moisture—approx. 0.45% (raw IBR did not contain any moisture), whereas SOL contained the most moisture (approx. 1.1%). Therefore, moisture content increased with increasing SOL in ASD. Under accelerated aging (40 ± 2 °C/75%RH ± 5% RH) and long-term (25 ± 2 °C/60%RH ± 5% RH) conditions, a slight increase in the moisture content was observed for each sample compared to the content at 0 time, i.e., the storage conditions did not increase the moisture adsorption of the tested materials.

The thermal stability of amorphous IBR under both storage conditions was approx. 10 °C lower compared to the crystalline form at 340–345 °C. SOL had reduced thermal stability (271–278 °C) compared to amorphous IBR, which promoted the increase in ASD degradation temperature with increasing IBR content. Thermal stabilities of the tested samples under both storage conditions were similar. Hence, the storage conditions did not significantly impact this parameter.

Regarding the generation of ASD, the SOL polymer exhibited favorable properties. Its high thermal stability makes it applicable to various technological processes without the risk of thermal degradation. During the tests, reflections in the XRPD diffractograms (Figure 6) were not observed, which would indicate diminished initiation of the API crystallization process. A slight increase in the moisture content during storage did not increase the molecular mobility of the API and promoted the formation of a physically stable ASD. The quench-cooling method used in this work produced an amorphous IBR with enhanced stability compared to that prepared by the solution evaporation method [28].

**Table 1:** The thermal decomposition parameters of measured samples.

Sample	Moisture Content [%]	I Stage of Decomposition				II Stage of Decomposition			
		Temp. Range [°C]	Mass Change [%]	T_m_ [°C]	Rate of Mass Loss [% min^−1^]	Temp. Range [°C]	Mass Change [%]	T_m_ [°C]	Rate of Mass Loss [% min^−1^]
**IBR raw**	0.00	353.9–447.8	89.20	427.9	17.31	-	-	-	-
**IBR_0**	0.37	329.9–447.1	88.52	428.7	18.18	-	-	-	-
**IBR_acc**	0.41	345.0–446.9	88.27	428.3	17.97	-	-	-	-
**IBR_long**	0.49	340.5–445.7	87.75	427.6	18.46	-	-	-	-
**IBR:SOL 1:1_0**	0.66	292.6–353.2	11.47	324.2	2.55	353.2–451.7	79.78	430.3	14.55
**IBR:SOL 1:1_acc**	0.97	289.2–348.4	12.19	325.7	2.81	348.4–451.4	79.48	429.8	13.57
**IBR:SOL 1:1_long**	0.94	282.0–350.2	12.38	323.9	2.81	350.2–452.3	78.03	429.9	13.81
**IBR:SOL 3:7_0**	0.92	284.3–356.0	16.90	325.8	3.65	356.0–454.5	76.53	430.8	12.79
**IBR:SOL 3:7_acc**	0.88	284.1–356.2	18.43	326.2	4.05	356.2–455.7	74.91	431.4	12.33
**IBR:SOL 3:7_long**	1.13	273.8–356.1	19.28	325.8	4.11	356.1–455.7	73.98	432.4	11.97
**IBR:SOL 1:9_0**	1.13	269.6–357.9	22.68	326.3	4.75	357.9–454.4	73.67	432.8	11.29
**IBR:SOL 1:9_acc**	0.93	279.2–361.5	22.16	326.5	4.63	361.5–458.0	71.65	434.9	11.59
**IBR:SOL 1:9_long**	1.27	275.3–361.7	21.29	326.0	4.32	361.7–458.3	71.19	431.7	11.32
**SOL_0**	1.10	276.4–358.5	23.60	326.7	5.01	358.5–457.7	71.54	433.3	10.91
**SOL_acc**	1.21	278.2–360.0	23.70	327.2	5.08	360.0–458.1	70.81	435.4	10.89
**SOL_long**	1.37	271.7–360.2	24.02	326.3	5.03	360.2–457.5	69.47	433.5	10.58

T_m_ temperature of maximum weight loss rate.

### 3.2. Powder X-ray Diffraction Analysis (XRPD)

The XRPD patterns of crystalline IBR, amorphous form of IBR, SOL, and formulations of amorphous IBR with Soluplus stored under accelerated (40 ± 2 °C/75%RH ± 5% RH) and long-term (25 ± 2 °C/60%RH ± 5% RH) conditions are shown in Figure 6. The raw IBR pattern showed intense characteristic peaks at the 2θ values of 5.8°, 13.4°, 13.8°, 16.3°, 18.4°, 19.1°, 21.5°, and 21.8° highlighting its crystalline nature. The recorded 2θ peak position corresponded to those reported for ibrutinib polymorph A [19,27,29]. The SOL diffractogram did not contain sharp peaks, but a characteristic halo indicated the amorphous nature of the polymer [30].

The lack of diffraction peaks in the XRPD pattern for the IBR sample prepared by the quench-cooling method confirmed the amorphousness of the substance obtained by this method. The same results were obtained after the stability tests.

The XRPD patterns for IBR:SOL ASD showed no reflections throughout the stability studies under both long-term and accelerated conditions. Therefore, amorphous IBR remained physically stable in the amorphous SOL matrix throughout the experiments.

### 3.3. Fourier Transform Infrared Spectroscopy (FTIR)

**Figure 7:**
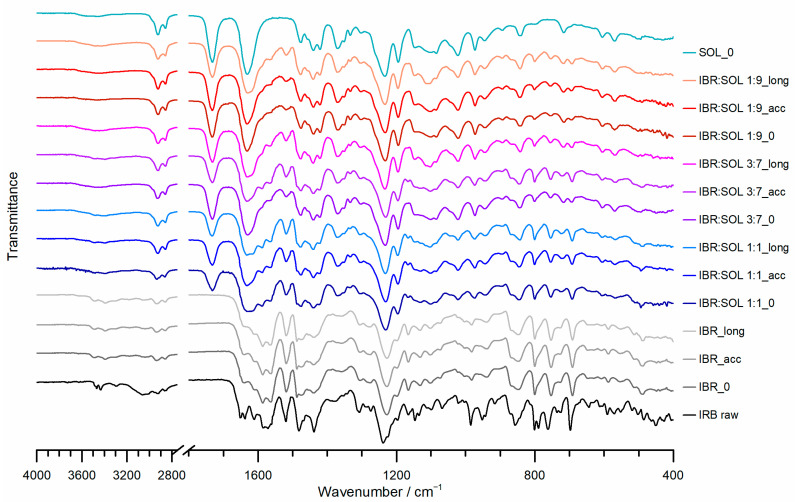
FTIR spectra of raw and amorphous IBR, Soluplus^®^, and IBR:SOL formulations at initial time and after stability tests.

The FTIR spectrum of raw IBR (Figure 7) showed several characteristic absorption peaks at 3468 cm^−1^, 3295 cm^−1^ (N-H stretching vibration), 1651 cm^−1^ (amide carbonyl C=O stretching vibration), 1611 cm^−1^, and 1519 cm^−1^ (C=C stretching vibration of the benzene ring). The obtained results were consistent with previous studies [18,31]. The FTIR spectrum of amorphous IBR (Figure 7), compared with crystalline form A, showed band broadening and reduced peak intensity, which was typical of the amorphous state owing to the destruction and disorganization of the crystal lattice.

Characteristic absorption peaks for Soluplus^®^ (Figure 7) were observed at 3447 cm^−1^ (O-H stretching), 2925 cm^−1^ (aromatic C-H stretching), 1732 cm^−1^, 1631 cm^−1^ (C=O stretching), and 1476 cm^−1^ (C-O-C stretching) [32].

The FTIR spectra of ASD IBR:SOL showed only bands related to IBR and SOL. However, certain peaks overlapped and, hence, could not be distinguished individually. The spectra of all formulations showed a broad band at 3200–3700 cm^−1^ stemming from the overlapping peaks of 3468 cm^−1^, 3295 cm^−1^ characteristic for IBR (N-H stretching vibration), and 3447 cm^−1^ characteristic for SOL (O-H stretching). Furthermore, the FTIR spectra of ASD showed an overlap of the C=O stretching bands for IBR at 1651 cm^−1^ and for SOL at 1631 cm^−1^ with the benzene ring C=C stretching bands at 1611 cm^−1^, related to IBR. The spectra of ASD IBR:SOL displayed bands at 1519 cm^−1^ (C=C stretching vibration of the benzene ring) for IBR, and the bands at 2925 cm^−1^ (aromatic C-H stretching), 1732 cm^−1^ (C=O stretching) and 1476 cm^−1^ (C-O-C stretching) for SOL (see Table S1 ESI). This proved that there was no interaction between the ASD components.

### 3.4. Scanning Electron Microscopy (SEM)

**Figure 8:**
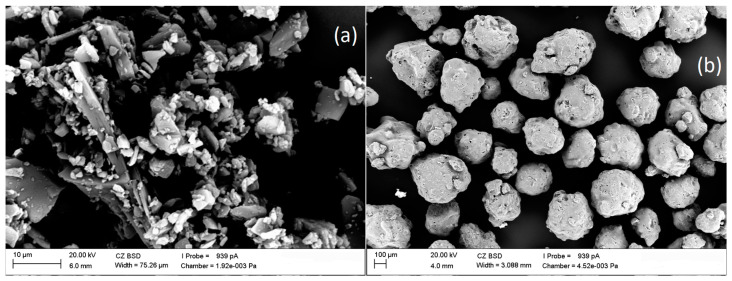
SEM images of (**a**) raw IBR and (**b**) Soluplus^®^, at initial time.

The samples of the amorphous form of IBR and its amorphous dispersions with SOL after preparing homogeneous mixtures were sieved using an 80 μm sieve. SEM images of the raw IBR and pure Soluplus^®^ are shown in Figure 8. Raw IBR appeared as a plate-shaped crystalline structure with sharp edges (Figure 8a). Soluplus^®^ showed irregular round-shaped particles with a rough and porous surface (Figure 8b).

**Figure 9:**
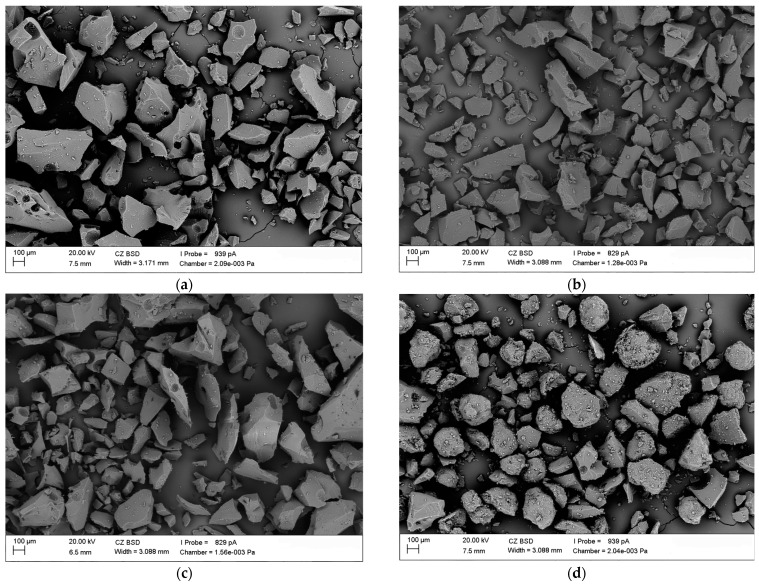

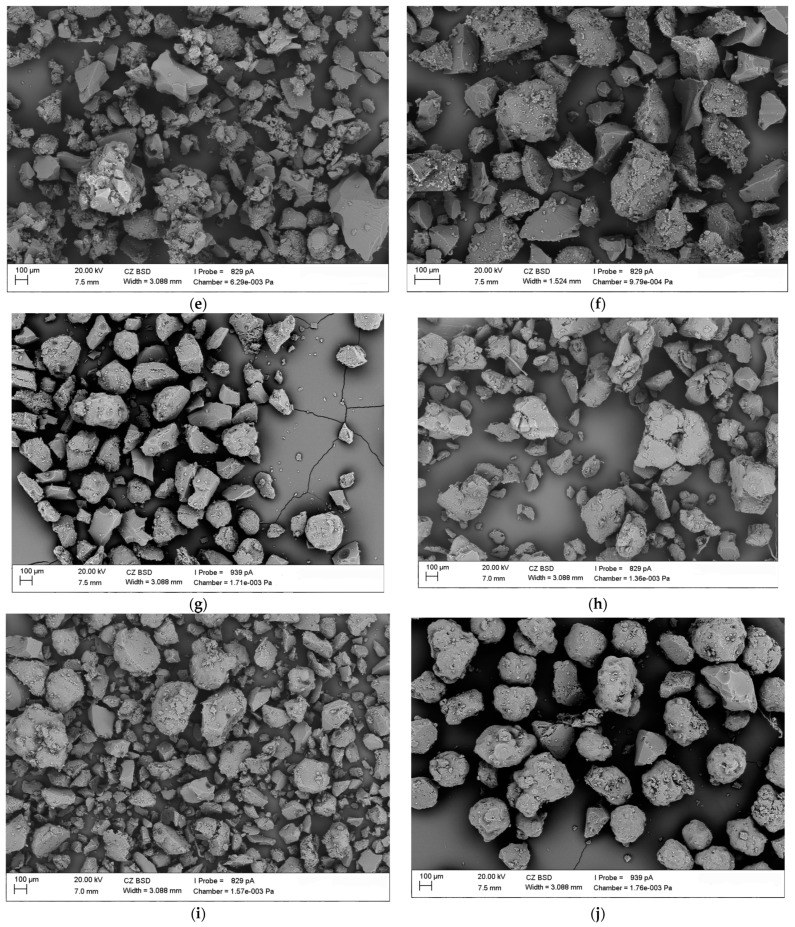

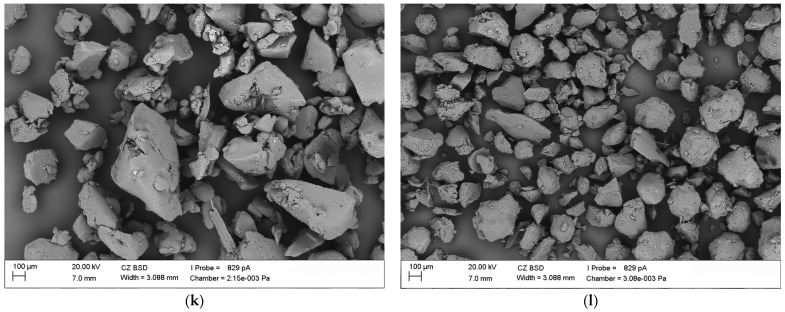
Ibrutinib SEM images of (**a**) amorphous IBR at initial time, (**b**) amorphous IBR stored for 6 months, (**c**) amorphous IBR stored for 12 months, (**d**) IBR:SOL 1:1 at initial time, (**e**) IBR:SOL 1:1 stored 6 months, (**f**) IBR:SOL 1:1 stored for 12 months, (**g**) IBR:SOL 3:7 at initial time, (**h**) IBR:SOL 3:7 stored for 6 months (**i**) IBR:SOL 3:7 stored for 12 months, (**j**) IBR:SOL 1:9 at initial time, (**k**) IBR:SOL 1:9 stored for 6 months, and (**l**) IBR:SOL 1:9 stored for 12 months.

The surface morphology of amorphous ibrutinib and ibrutinib–Soluplus formulations were obtained using SEM immediately after preparation, after 6 months under accelerated conditions (40 ± 2 °C/75%RH ± 5% RH), and after 12 months under long-term conditions (25 ± 2 °C/60%RH ± 5% RH). The stability tests are shown in Figure 9. IBR:SOL formulations showed irregular, round-shaped particles and aggregation, creating a larger particle size. Upon preparation of ASD, amorphous IBR was uniformly distributed in the matrix of the amorphous polymer. No significant differences in particle size and shape were observed in the samples stored under accelerated and long-time conditions.

### 3.5. Physical Stability Studies

The main problem with ASDs is their tendency to crystallize during storage and the effect of moisture, which increases the molecular mobility of drugs and promotes crystallization [33]. The presence of water in a system increases the free volume, therefore plasticizing the system and resulting in increased mobility [34,35,36]. It has been demonstrated that water sorption affects the physical stability of both single and binary amorphous systems. The increase in water content can accelerate the crystallization process in amorphous sucrose and glucose [37]. This is attributed to reduced viscosity, which increases the probability of nucleation and subsequent crystallization. For amorphous lactose, an increase in water sorption can result in a progressive decrease in the peak crystallization temperature [38]. Reports have shown that the enhanced affinity of a polymer for water is associated with a reduction in its ability to inhibit drug crystallization [39].

Morrow et al. analyzed the effect of the amorphization method on physicochemical properties, including moisture content, of amorphous sucrose prepared by freeze-drying, spray drying, ball milling, melt quenching, and spin melt quenching. The results indicated that samples prepared by freeze-drying and spray-drying exhibited higher moisture content (2.14% and 3.16%), lower glass transition temperature (47.92 °C for freeze-drying and 39.78 °C for spray-drying), and the absence of thermal decomposition indicator compounds. Conversely, samples prepared by melt quenching and spin melt quenching exhibited a lower moisture content (0.14% and 0.55%), a higher glass transition temperature (64.92 °C for melt quenching and 59.20 °C for spin melt quenching), and the presence of thermal decomposition indicator compounds. These general differences were attributed to differences in the route of amorphization, as samples prepared by freeze-drying and spray drying were in solution, whereas samples prepared by melt quenching and spin melt quenching were by heating crystalline sucrose to form a melt. In terms of physicochemical properties, ball milling exhibited characteristics that were intermediate between those of the amorphous sucrose prepared in solution and those of the sucrose prepared by melt amorphization. It exhibited an intermediate moisture content (1.00%) and glass transition temperature (59.32 °C) and contained small but significant amounts of thermal decomposition indicator compounds [40].

Despite the presence of absorbed water in the stored samples, the molecular mobility of IBR in the prepared matrixes was slow enough to avoid crystallization, even when stored under accelerated conditions. This revealed the physical strength of these polymeric systems in the presence of high humidity and temperature.

Amorphous ibrutinib and IBR-SOL formulations stored for 6 months under accelerated conditions (40 ± 2 °C/75%RH ± 5% RH) and 12 months under long-term conditions (25 ± 2 °C/60%RH ± 5% RH) were physically stable throughout the test.

The physical stability studies of amorphous solid dispersions of ibrutinib were previously reported [10]. To physically stabilize this drug, polymers polyvinylpyrrolidone (PVP), Soluplus^®^, and poly(vinyl pyrrolidone-vinyl acetate) copolymer (PVPVA) were combined with API by Hot-Melt Extrusion. The ibrutinib samples crystallized after 3 months at accelerated conditions, whereas under long-term conditions, the samples remained amorphous for 6 months. When formulated (preliminary IBR formulation), the physical stability of IBR was lower, and IBR crystallized only after 1 month at 40 °C/75%RH, and after 6 months at 25 °C/60%RH.

## 4. Conclusions

For the first time, an amorphous ibrutinib was successfully obtained using the quench-cooling method with very high process efficiency. Also, different formulations of amorphous active pharmaceutical ingredient (API) with Soluplus (SOL) in various weight ratios, 1:9, 3:7, and 1:1, respectively, were prepared.

The lack of significant interactions between the ingredients of the formulation was confirmed by FTIR analysis.

Amorphous ibrutinib and the IBR-SOL formulations were stored in two environmental simulation chambers, 25 ± 2 °C/60%RH ± 5% RH (long-term conditions, 12 months) and 40 ± 2 °C/75%RH ± 5% RH (accelerated conditions, 6 months) and were found to be physically stable throughout the study.

The thermal stabilities of the tested samples under both storage conditions were similar and relatively high. Therefore, the storage conditions did not significantly impact this parameter. The high thermal stability makes it possible to use various technological processes without the risk of thermal degradation.

## Data Availability

The original contributions presented in the study are included in the article/supplementary material, further inquiries can be directed to the corresponding author/s.

## Supplementary Materials

The following supporting information can be downloaded at: https://www.mdpi.com/article/10.3390/polym16141961/s1. Table S1: Characteristic Fourier transform infrared spectroscopy (FTIR) bands for IBR, SOL and IBR:SOL formulations.
