# Supplementary material for: Stability Studies of Amorphous Ibrutinib Prepared Using the Quench-Cooling Method and Its Dispersions with Soluplus®
Source: Polymers (Basel). 2024 Jul 9;16(14):1961. doi: 10.3390/polym16141961 (PMC11280989; doi:10.3390/polym16141961)
Supplement: Supplementary file 1 [file polymers-16-01961-s001.zip › polymers-3034945-supplementary.pdf]

SUPPORTING INFORMATION

Stability studies of amorphous ibrutinib prepared using the quench cooling method and its dispersions with Soluplus®

Igor Mucha [1], Bożena Karolewicz [2,*] and Agata Górniak [3]

[1] Department of Basic Chemical Sciences, Wroclaw Medical University, Borowska 211 A, 50-556 Wroclaw, Poland; igor.mucha@umw.edu.pl

[2] Department of Drug Form Technology, Wroclaw Medical University, Borowska 211 A, 50-556 Wroclaw, Poland; bozena.karolewicz@umw.edu.pl

[3] Laboratory of Elemental Analysis and Structural Research, Wroclaw Medical University, Borowska 211 A, 50-556 Wroclaw, agata.gorniak@umw.edu.pl

* Correspondence: bozena.karolewicz@umw.edu.pl; Tel.: +48 71 78 40 317

Table S1. Characteristic Fourier transform infrared spectroscopy (FTIR) bands for IBR, SOL and IBR:SOL formulations.

vibrations type	IBR	IBR_0	IBR_acc	IBR_long	IBR:SOL 1:1_0	IBR:SOL 1:1_acc	IBR:SOL 1:1_long	IBR:SOL 3:7_0	IBR:SOL 3:7_acc	IBR:SOL 3:7_long	IBR:SOL 1:9_0	IBR:SOL 1:9_acc	IBR:SOL 1:9_long	SOL	SOL_acc	SOL_long
C=C	1519	1519	1518	1518	1518	1519	1519	1518	1519	1518	1517	1519	1518	-	-	-
C-O-C	-	-	-	-	1475	1475	1477	1476	1475	1477	1475	1475	1477	1476	1477	1477
C=O	-	-	-	-	1732	1734	1732	1732	1732	1731	1732	1732	1732	1732	1732	1731
C-H	-	-	-	-	2926	2928	2926	2925	2924	2925	2924	2924	2924	2925	2924	2924
